# 

**Published:** 1895-08-03

**Authors:** 


### CONTENTS.

**Medical Politics in Yorkshire** . . . . . . . . 299

**Pay Hospitals: Fiat Experimentum** . . . . . 300

**The Life History of Aneurisms.** By A. Pearce Gould, F.R.C.S. . . . . . . . . . . . 301

**The Speech of Souls** . . . . . . . . . . 302

**Annotations.**—Insanity in the Medical Profession—A Practical Aspect of Isolation—Our Indian Troops . . . . . 303

**Notes and News** . . . . . . . . . . . 304

**Medical Progress and Hospital Clinics—**

British Medical Association.—The Presidential Address . 305

Progress in Medicine.—Disease of the Respiratory System (*continued*) . . . . . . . . . . 306

Progress in Surgery.—Cerebral Surgery (*continued*)—Fractures and Dislocations (*continued*) . . . . . . 307

New Appliances and Things Medical . . . . . 310

**The Institutional Workshop—**

British Medical Association.—Annual Museum . . . 311

### EXTRA SUPPLEMENT—THE NURSING MIRROR.

**News from the Nursing World**—The Colours of our Princess—Personal Recognition—Help Well Bestowed—"Can't Afford It."—Girl Lectures—A Novel Forecast—An Opinion Worth Attention—Progress at Kilmarnock—Those Uniforms Again—A Grateful Patient—The Bishop of London—Short Items . . cxix

**The Royal National Pension Fund for Nurses** . . cxxii

**Princess's Colours** . . . . . . . . . . cxxv

**Elementary Anatomy and Surgery for Nurses.** By W. McAdam Eccles, M.D., M.S., F.R.C.S. XXVII.—Burns and Scalds . . . . . . . . . . . . cxxi

**Appointments** . . . . . . . . . . . cxxv

**Where to Go** . . . . . . . . . . . cxxv

**Everybody's Opinion** . . . . . . . . . cxxvi

**Royal British Nurses' Association** . . . . . cxxvii

